# Entropy-dependent human motor modulation consistent with morphological computation in a single subject

**DOI:** 10.3389/frobt.2026.1734848

**Published:** 2026-02-10

**Authors:** Tsubasa Wakatsuki, Norimasa Yamada

**Affiliations:** 1 Institute of Engineering, Tokyo University of Agriculture and Technology, Koganei, Japan; 2 Faculty of Health and Sport Sciences, Chukyo University, Toyota, Japan

**Keywords:** coefficient of variation, embodied intelligence, foreperiod, low pass filter, motor control, reaction time, shannon entropy, temporal uncertainty

## Abstract

Morphological computation (MC)—the idea that body mechanics contribute to computation—has been widely explored in robotics and examined in humans from a physiological perspective. In this study, we report a behavioral pattern consistent with MC under temporal uncertainty. This proof-of-concept single-subject study examined whether human motor control shows behavioral signatures consistent with MC within a temporal-preparation paradigm. One participant completed 160 trials across four entropy levels (0, 1.0, 1.5, 2.0 bits) in two tasks: a low-embodiment button-pressing movement and a high-embodiment reaching movement. The reaching movement tended to show decreasing response variability (coefficient of variation, CV) with increasing temporal uncertainty, whereas the button-pressing movement tended to remain flat or slightly increase. Reaction time (RT) patterns also diverged: RTs tended to lengthen with longer foreperiods in the reaching condition but shortened in the button-pressing movement. Moreover, spatial accuracy in the reaching movement tended to improve across foreperiods. These adaptations emerged without explicit strategy instructions, may reflect sensitivity to temporal context. Taken together, these patterns appear consistent with MC-inspired accounts in which limb mechanics and modest co-contraction may filter temporal uncertainty rather than amplify it. Although constrained by a single-subject, four-level design, the findings offer preliminary evidence that is suggestive of embodied-intelligence principles that may generalize to human motor control, highlighting commonalities between biological and robotic systems in brain–body–environment dynamics.

## 1 Introduction

### 1.1 Morphological computation theory and experimental gap

The concept of morphological computation (MC) originates from robotics and proposes that the properties of the physical body can perform computational functions traditionally attributed to neural processing (Pfeifer and Bongard, 2006). This paradigm suggests that intelligence emerges from dynamic interactions among the brain, the body, and the environment, rather than from centralized neural processing alone (Chiel and Beer, 1997). Classic examples include passive dynamic walkers that achieve stable locomotion through mechanical interactions without active control (McGeer, 1990), compliant quadruped locomotion that self-stabilizes via body dynamics (Iida et al., 2005), and soft robotic systems that explicitly leverage body compliance for stability and control (Rus and Tolley, 2015; Trivedi et al., 2008). Recent reviews have synthesized MC as a design principle for embodied intelligence and have highlighted open challenges in turning morphology-enabled information processing into quantitative tools (Hauser and Hughes, 2024; Zhao et al., 2024). Similarly, information-theoretic measures have been proposed to quantify the contribution of morphology in the sensorimotor loop (Zahedi and Ay, 2013; Ghazi-Zahedi and Rauh, 2015).

Despite these advances, MC in biological systems remains largely untested at the behavioral level. Computational studies using information-theoretic measures applied to human biomechanical models have reported higher MC in more complex muscle models (Ghazi-Zahedi et al., 2016; Haeufle et al., 2020), but these studies have relied on simulations rather than measurements from experimental human movements. In addition to such model-based approaches, recent studies have examined upper-limb coordination and human–robot cooperative tasks from an MC- or information-theoretic perspectives. Wang et al. (2023) used a neuromusculoskeletal model of a person playing octaves on a piano to analyze how upper-limb mechanics and control policies shape complex sequential movements, and Wang et al. (2024) assessed human–robot cooperative piano performance using an entropy-based evaluation of cooperation quality. These studies suggest that morphology and control matter under interaction-induced uncertainty because cooperative performance inherently exposes each partner to uncertainty about the other’s timing, dynamics, and contribution. The results of experiments with human participants have also shown that limb impedance and co-contraction are actively regulated under sensory uncertainty and interaction demands (Carboni et al., 2021; Börner et al., 2023) and can be computationally optimized to improve information use (Cheng et al., 2025). In this study, we provide a simple behavioral paradigm that isolates the impacts of morphology on human motor behavior under controlled temporal uncertainty.

### 1.2 Information-theoretic approach to temporal preparation

From an MC perspective, situations in which the timing of the “go” signal is uncertain are especially informative because they reveal how the body may help to handle temporal uncertainty. The foreperiod paradigm (Klemmer, 1957) provides a standard framework for studying such temporal preparation. In tasks with a variable foreperiod, greater uncertainty as to the foreperiod is associated with longer reaction times (RTs) and increased trial-to-trial variability—the classic foreperiod effect—which is typically interpreted as reflecting central predictive timing and attention, with the motor periphery considered as a passive executor. Within this framework, we applied planned entropy (Shannon, 1948) to manipulate prior temporal uncertainty in a principled manner.

Pressing a button is a minimally embodied action, whereas reaching involves coordination among multiple joints, in which the arm’s mechanical properties such as mass distribution, joint compliance, and muscle viscoelasticity can plausibly contribute to filtering-like stabilization (Haeufle et al., 2020). If the body’s mechanical properties can be considered to perform computational functions in some sense, then they may attenuate initiation variability under higher temporal uncertainty rather than simply transmitting central noise, which would effectively act as a low-pass filter for temporal uncertainty. Therefore, a temporal preparation paradigm with an extremely simple motor task and entropy-controlled foreperiods offers a direct behavioral test of whether morphology contributes computationally to motor preparation and initiation.

### 1.3 Present study

Building on this mechanistic foundation, we considered whether a behavioral functional signature of morphology under temporal uncertainty can be isolated in a classical variable-foreperiod framework. Using an information-theoretic manipulation of prior temporal uncertainty (planned foreperiod entropy), we directly compare a minimally embodied button-pressing movement with a highly embodied reaching movement for each the same participant.

Computational and biomechanical studies have suggested that morphology can provide filtering-like stabilization via mass distribution, joint compliance, and viscoelasticity (Haeufle et al., 2020). Accordingly, we expected that reaching would show a decrease in the variability of reaction times (coefficient of variation, CV) as uncertainty increased, whereas pressing a button would remain flat or increase slightly to reflect a more direct neural-to-motor translation. We further examined trends in mean reaction time (RT) across foreperiods to test for effector-specific differences in triggering strategies.

The key contribution of this work is to quantify what morphology accomplishes behaviorally under uncertainty, that is, a qualitative inversion of RT stability (CV) across effectors within a given individual under identical entropy-controlled foreperiods, together with effector-specific foreperiod–RT trends. This provides a concise target for multimodal follow-ups—co-recording electromyography (EMG), kinematics/EEG—to partition passive morphology from actively modulated impedance. In summary, we extend on classical works on foreperiod modeling in two ways. First, we move beyond mean RT costs to also consider stability, which is quantified as the coefficient of variation (CV). Second, we go beyond a single-effector button press and instead compare two movements with different degrees of embodiment within the same participant. As a result, we obtained a compact behavioral signature that is consistent with MC-inspired accounts based on embodied intelligence.

## 2 Methods

### 2.1 Experimental design and participant

One right-handed male graduate student (age: 24 years, height: 163 cm, weight: 54 kg, shoulder–elbow length: 25 cm, elbow–wrist length: 24 cm) participated in this study after providing informed consent. The participant had no notable athletic experience. The study involving human participants received ethical approval from the Ethics Review Board of Tokyo University of Agriculture and Technology (Permit No. 251203-0748), and all methods were performed in accordance with relevant guidelines and regulations.

This proof-of-concept study adds human behavioral evidence consistent with morphological computation. MC is theoretically an idiosyncratic phenomenon, deeply dependent on individual physical characteristics such as limb length, mass, and joint flexibility. We prioritized a high-resolution within-participant design to avoid obscuring embodiment-specific patterns that may arise when prematurely averaging across heterogeneous morphologies. Therefore, to rigorously test for the existence of this phenomenon, we adopted a single-subject design that allowed for an intra-individual comparison between low-embodiment (button-pressing) and high-embodiment (reaching) conditions under controlled uncertainty. The primary focus of this study was not to assess the generalizability of the results, but rather to establish a clear behavioral pattern consistent with theory in a single participant.

### 2.2 Experimental apparatus and task

#### 2.2.1 Variable-foreperiod task

**FIGURE 1:**
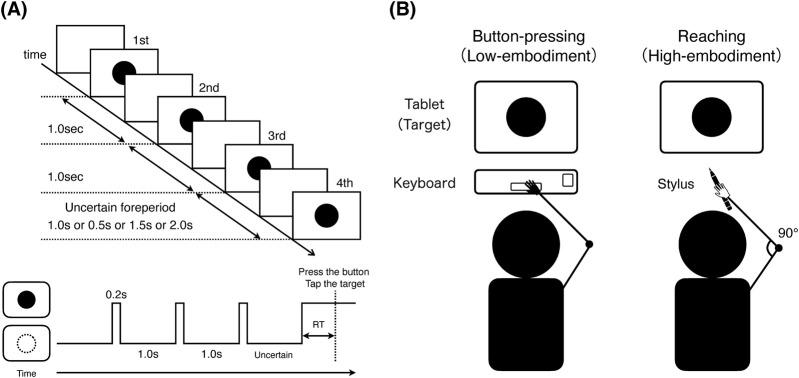
Experimental task and movement conditions. **(A)** Trial sequence under graded temporal uncertainty (four foreperiods = 0.5, 1.0, 1.5, 2.0 s). Each block implements a planned foreperiod probability distribution that sets the temporal entropy (0, 1.0, 1.5, 2.0 bits). Reaction time (RT) was defined as the interval from the onset of the fourth target to the participant’s response. In the button-pressing condition, the response was pressing the spacebar; in the reaching condition, it was tapping the target with the stylus. **(B)** Movement conditions. Button-pressing: minimally embodied spacebar response. Reaching: stylus tap with a 90° elbow-flexion start posture to preserve ecological validity.

The participant performed a foreperiod paradigm where a target (red circle) appeared intermittently four times on a 60 Hz tablet display (iPad Air, 13 inch, Model MCNT4J/A, Apple Inc.), and the participant responded to the fourth presentation (Figure 1A, top). In both movements (Section 2.2.2), the targets were red circles with a radius of 6.1 cm, presented at the center of a white background. The task was executed using PsychoPy (v2021.2.3, University of Nottingham). The display was mirrored to a tablet to record tap-location data.

The first three intervals between target presentations were fixed at 1.0 s to establish temporal expectation, while the critical foreperiod (the interval between the third and fourth presentations) was systematically manipulated to create varying levels of temporal uncertainty (see 2.2.3). RTs were displayed in real time to motivate rapid responses, and premature responses (responses made before the fourth stimulus presentation) triggered immediate block repetition, ensuring adherence to temporal constraints. This response to premature reactions is in accordance with Klemmer (1957). Participants received no information about foreperiod duration manipulation or temporal uncertainty (entropy manipulation) and were simply instructed to “respond to the fourth stimulus as quickly as possible.”

#### 2.2.2 Movement conditions

Button-pressing condition (Figure 1B Left): Participants pressed the spacebar on a standard keyboard using their dominant hand index finger—a classical style representing minimal embodiment where neural commands directly control simple mechanical displacement.

Reaching condition (Figure 1B Right): Participants were seated at a distance where a stylus held naturally in their extended arm would contact the tablet screen. Trials began from a posture with the elbow flexed approximately 90° from this natural extension position, requiring participants to perform goal-directed reaching movements by extending their elbow to tap the target with the stylus. This setup engaged multiple joints and the arm’s mechanical properties—mass distribution, joint compliance, and muscle viscoelasticity—contrasting with the simple finger movement required for button-pressing.

#### 2.2.3 Entropy manipulation

Entropy is determined by the number of events and their occurrence probabilities. In this study, the events refer to foreperiod durations, with 1.0 s serving as the baseline. Four durations were used: 0.5 s (shorter than baseline), 1.0 s (baseline), 1.5 s (longer than baseline), and 2.0 s (substantially longer than baseline). Temporal uncertainty was manipulated through Shannon entropy (Shannon, 1948):

$$H=-\Sigma \;pᵢ\;\log_{2}\;pᵢ$$

where pᵢ represents the probability of each foreperiod duration. With four events, the maximum entropy—a completely random task where four foreperiod durations occur at 25% each—is 2.0 bits. Conversely, the minimum entropy—a task where one event (1.0 s) occurs with certainty (100%)—is 0 bits. In addition to these two entropy levels, 1.0 bit- and 1.5 bit-tasks were created by adjusting the occurrence probabilities. Four entropy conditions (0, 1.0, 1.5, and 2.0 bits) were implemented with their specific probability distributions detailed in Supplementary Table S1.

#### 2.2.4 Session structure and randomization

Each experimental session comprised eight blocks of 20 trials (160 total trials), counterbalanced across two movement conditions and four entropy levels. The order of condition blocks was randomized using the RandomSample function in Mathematica 12.2 (Wolfram Research, IL, United States).

### 2.3 Data analysis

#### 2.3.1 Reaction time

For the button-pressing condition, RT was defined as the elapsed time from the onset of the fourth target to the spacebar press. For the reaching condition, RT was defined as the elapsed time from the onset of the fourth target to the moment the stylus contacted the target (Figure 1A, bottom). Thus, the reaching RT includes movement time. Because we do not compare absolute RT values between the button-press and reaching conditions, this definitional difference does not affect our analyses. The relationship between entropy level and RT was assessed using linear regression:

$$RT=\beta_{0}+\beta_{1}\times Entropy+\epsilon$$

where $\beta_{0}$ is the intercept, $\beta_{1}$ represents the slope coefficient, and ϵ is the error term.

#### 2.3.2 Response stability

Response stability was quantified using the CV, a normalized measure of variability:

$$CV=\frac{\sigma_{RT}}{\mu_{RT}}\times 100$$

where $\sigma_{RT}$ and $\mu_{RT}$ are the standard deviation and mean RT for each entropy condition, respectively. The relationship between entropy level and the CV was assessed using linear regression:

$$CV=\beta_{0}+\beta_{1}\times Entropy+\epsilon$$

where $\beta_{0}$ is the intercept, $\beta_{1}$ represents the slope coefficient, and ϵ is the error term. Pearson correlation coefficients (r) were calculated to quantify the strength and direction of the relationship.

#### 2.3.3 Foreperiod duration

Linear regression analysis was used to examine the relationship between foreperiod duration and RT:

$$RT=\beta_{0}+\beta_{1}\times ForeperiodDuration+\epsilon$$

where $\beta_{0}$ is the intercept, $\beta_{1}$ represents the slope coefficient, and ϵ is the error term.

#### 2.3.4 Spatial accuracy

Spatial accuracy was assessed by calculating the Euclidean distance from each tap location to the target center (0, 0):

$$Euclidean\;distance=\sqrt{{\left(Touch_{X}\right)}^{2}+{\left(Touch_{Y}\right)}^{2}}$$

where $Touch_{X}$ and $Touch_{Y}$ represent the horizontal and vertical coordinates of the tap position, respectively.

#### 2.3.5 Statistical analysis

Following common outlier-handling practices in reaction-time research (*cf.* Anscombe and Guttman, 1960), trials with RTs falling outside the blockwise mean ±2 SD (within each Movement × Entropy block) were excluded. This trimming was applied once (non-iteratively), and all analyses used the remaining trials. All tests were two-sided with α = 0.05. We report exact p-values together with effect sizes and 95% confidence intervals (CIs). Linear trends for entropy–RT and foreperiod–RT were estimated at the trial level using ordinary least squares (OLS), treating each trial as one observation. The entropy–CV relationship was analyzed at the condition level (four entropy means) using OLS. LinearModelFit and ANOVA function in Mathematica were used for the regression and ANOVA analyses. For spatial accuracy, a one-way ANOVA tested differences in Euclidean distance to the target center across four foreperiod durations (0.5, 1.0, 1.5, 2.0 s), with *η* ^*2*^ reported as the effect size.

Effect sizes for correlation differences between movement conditions were calculated as

$$\Delta r=\left|r_{reaching}-r_{button-pressing}\right|$$

where r denotes the Pearson’s correlation coefficient. Cohen’s conventions were applied to interpret effect sizes, with Δr>0.1 considered small effects, Δr>0.3 considered medium effects, and Δr>0.5 considered large effects (Cohen, 1988).

We emphasize effect sizes and 95% CIs given the small number of condition means (four levels; df = 2), where null-hypothesis tests have limited power. Exact statistics for all models (coefficients, n, df, *t*, *p*, *r*, 95% CIs, *R* ^*2*^) are reported in the Supplementary Material.

## 3 Results

### 3.1 Valid trials and post-calculated entropy

Trials with RTs exceeding ±2 standard deviations were removed, leaving 75/80 valid trials in button-pressing and 76/80 in reaching (overall retention 94.4%). Because entropy depends on the number and probability of foreperiod durations, preset and post-calculated entropy differed slightly; valid trial counts and post-calculated entropy for each block are shown in Supplementary Table S2.

### 3.2 Effects of entropy on RT and its CV

**FIGURE 2:**
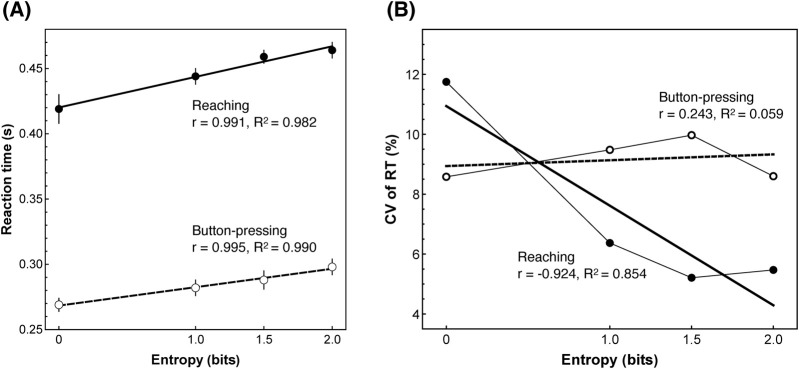
Planned entropy analysis of RT and CV. In both figures, black markers indicate reaching condition and white markers indicate button-pressing condition; error bars show standard errors. The solid line is the regression for reaching, and the dashed line is the regression for button-pressing. **(A)** Correlation between entropy and reaction time (RT). RT increases with entropy in both movement conditions. These results support Klemmer’s (1957) findings, indicating that the present experiment was properly implemented. **(B)** Correlation between entropy and the coefficient of variation (CV) of RT. Button-pressing shows a tendency for CV to increase with entropy, whereas reaching shows a decrease in CV as entropy increases.

Figure 2A shows the changes in RT. Linear regression confirmed that RT increased with temporal entropy in both conditions. Parallel analyses using post-calculated entropy showed comparable patterns. Detailed statistics are provided in Supplementary Table S3.

The central finding is a divergence in how uncertainty maps onto variability across movement conditions (Figure 2B). Button-pressing showed no reliable entropy–CV relationship (*r* = 0.243, *p* = 0.759). In stark contrast, the reaching movement showed a clear negative association between entropy and CV. Although this trend did not reach conventional statistical significance (p = 0.076), the effect size was large (r = −0.924, 95% CI [-0.998, 0.331]), consistent with a strong decrease in response variability as temporal uncertainty increased. Parallel analyses using post-calculated entropy showed a similar pattern (see Supplementary Figure S1). Given the limited power, formal inference was constrained; nevertheless, the between-condition correlation difference was very large (planned entropy: *Δr* = −1.167, *|Δr|* = 1.167; post-calculated entropy: *Δr* = −0.978, *|Δr|* = 0.978) and the patterns were systematic. Both conditions exhibited mild non-monotonicity at maximum entropy. Detailed statistics are provided in Supplementary Table S4.

### 3.3 Foreperiod duration effects on RT and spatial accuracy

**FIGURE 3:**
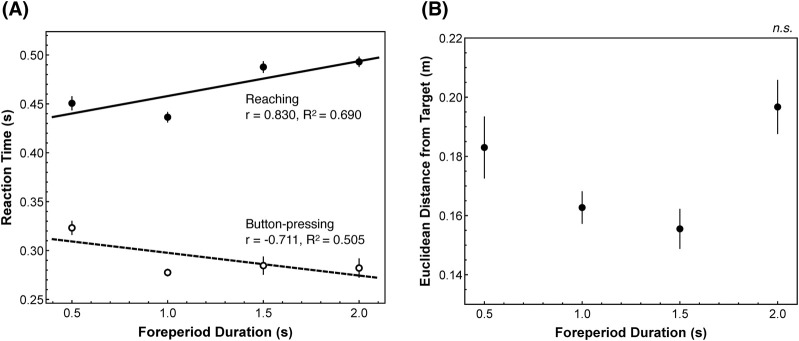
Foreperiod analysis of RT and spatial accuracy. **(A)** Correlation between foreperiod and reaction time (RT). Black markers indicate reaching condition and white markers indicate button-pressing condition; error bars show standard errors. The solid line is the regression for reaching, and the dashed line is the regression for button-pressing. In the button-pressing condition, RT decreased as the foreperiod increased, supporting the well-known variable-foreperiod effect reported in prior studies; by contrast, the reaching condition showed the opposite pattern. **(B)** Relationship between foreperiod and spatial accuracy in reaching. This figure shows the Euclidean distance from the target center to the tap location. Error bars indicate the standard error. Accuracy improved progressively from 0.5 s to 1.5 s, consistent with the result in **(A)** under the speed–accuracy trade-off.

Analysis of foreperiod duration effects revealed contrasting patterns between movement conditions (Figure 3A). In the button-pressing condition, a non-significant negative correlation (*p* = 0.288) was observed between foreperiod duration and RT, with a large negative effect size (*r* = −0.711, 95% CI [-0.993, 0.790]). By contrast, reaching showed a non-significant positive correlation (*p* = 0.169), indicating longer RTs with a longer preparation time, with a large positive effect size (*r* = 0.830, 95% CI [-0.648, 0.996]). The difference in correlations was descriptively large (*Δr* = 1.541, *|Δr|* = 1.541). Detailed statistics are provided in Supplementary Table S5.

Additionally, spatial accuracy showed systematic variation in the Euclidean distance from the target center across foreperiod durations (Figure 3B). Excluding the 2.0 s foreperiod, accuracy improved progressively from 0.5 s to 1.5 s. However, a one-way ANOVA revealed no significant effect of foreperiod duration on spatial accuracy (*F*(3, 71) = 1.334, *p* = 0.270, *η* ^*2*^ = 0.053).

## 4 Discussion

The observed reduction in CV with increasing temporal entropy suggests a contribution of MC, whereby limb mechanics attenuate uncertainty-related trial-to-trial variability in initiation. In addition, the reaching-specific inversion of the foreperiod–RT function—absent in button-pressing—supports an embodied intelligence interpretation. While confirmatory multi-participant studies are required, these results offer a proof-of-concept demonstration that, in humans, adaptation to temporal uncertainty can be partly realized through the body’s own mechanics.

### 4.1 Proof-of-concept behavioral evidence of MC in humans

As an internal validity check, the positive entropy–RT slopes replicated Klemmer’s (1957) foreperiod-uncertainty effect in direction and magnitude for the planned-entropy (primary) analyses across both effectors. Post-calculated entropy showed convergent positive slopes, with significance for button-pressing and a near-threshold trend for reaching, reflecting limited power with four levels (df = 2). Together, this indicates that the temporal-uncertainty manipulation and task implementation functioned as intended. Our findings provide proof-of-concept behavioral evidence consistent with MC in human motor control. Specifically, the button-pressing movement showed a small positive entropy–CV slope as planned entropy increased, whereas the reaching movement showed a clear decrease in CV with increasing planned entropy. While this effect did not reach conventional significance, its direction was consistent with our hypothesis, and its magnitude—as indicated by the very large effect size (r = −0.924)—suggests a meaningful reduction in variability under higher uncertainty for the reaching movement. This pattern supports the interpretation that limb mechanics attenuate uncertainty-related trial-to-trial variability in a filtering-like manner, thereby stabilizing motor initiation under uncertainty without committing to a specific anatomical mechanism.

In this study, prior temporal uncertainty refers to the experimentally manipulated planned entropy. Trial-to-trial variability in response initiation was summarized by CV. Accordingly, the decrease in CV in the reaching movement with increasing uncertainty indicates less initiation variability under higher prior uncertainty when the effector is highly embodied. This interpretation aligns with embodied-intelligence views in which computation is distributed across neural and mechanical elements that adapt to environmental context (Pfeifer and Bongard, 2006). For conceptual grounding from robotics, passive-dynamic walkers and compliant soft-robotic systems illustrate how mechanical structure can provide stability and simplify control (e.g., McGeer, 1990; Iida et al., 2005; Trivedi et al., 2008; Rus and Tolley, 2015). Analogously, our data suggest that the arm’s mechanical properties may serve as a computational resource that filters—rather than amplifies—temporal uncertainty.

### 4.2 Mechanistic insights into embodied motor control

As established in Section 4.1, higher prior temporal uncertainty tended to reduce CV in the reaching movement. Here, we examine how foreperiod duration modulated mean RT and spatial precision, revealing a context-dependent shift in triggering strategy and—as shown later—divergent foreperiod effects across effectors. In the button-pressing movement, mean RTs tended to shorten as the foreperiod increased—a trend directionally consistent with the classic variable-foreperiod effect (Klemmer, 1957; Niemi and Näätänen, 1981)—with a large negative effect size (*r* = −0.711). In the reaching movement, the relation showed the opposite trend. Mean RTs tended to lengthen with longer foreperiods, with a large positive effect size (*r* = 0.830). A purely central-state account—attributing the pattern solely to attention/arousal/expectancy—would typically predict similar foreperiod–RT trends across effectors. Therefore, the effector-specific inversion suggests that any central changes are channeled through and shaped by limb mechanics and control, indicating context-sensitive adjustment of the triggering strategy at the effector level.

The spatial accuracy results converge on the same interpretation. From 0.5 s to 1.5 s, the reaching movement showed a consistent trend toward improved precision despite no accuracy instructions, consistent in direction with the classic speed–accuracy trade-off (Woodworth, 1899). If movements had been executed identically across foreperiods, accuracy would have remained constant; instead, the graded improvement implies that the motor system reallocates preparation to match the available time with shorter intervals biasing faster/less precise responses and longer intervals allowing slower/more precise responses. The 2.0 s foreperiod deviated: RTs were slower while precision declined relative to 1.5 s, contrary to the classic speed–accuracy trade-off. Given the fewer trials at this foreperiod by design, we treat this as hypothesis-generating, suggesting that preparation benefits may saturate or degrade beyond a bounded temporal window.

### 4.3 Toward an embodied intelligence framework

The convergence of our two primary findings supports a broader view of embodied intelligence, in which morphology and impedance control jointly shape behavior under temporal uncertainty. In our data, prior temporal uncertainty modulated both the CV of RT in a movement-dependent manner and the foreperiod–RT function, and these temporal effects were coupled with systematic changes in spatial accuracy. This pattern is consistent with the idea that higher-level expectations about timing are implemented through effector-specific triggering strategies that exploit the mechanical properties of a limb. Rather than treating the body as a passive channel, this perspective regards morphology and impedance as tunable resources that can stabilize performance under uncertainty, while still allowing flexible control.

An inertia-only account would treat lower variability as a by-product of fixed limb mass and inertia. However, within the same participant, we observed a sign inversion of the foreperiod–RT relation across effectors: button pressing showed the classic variable-foreperiod pattern (longer foreperiods associated with shorter RT), whereas reaching showed the opposite trend, and systematic gains in spatial accuracy with longer foreperiods despite no accuracy instruction. This combination of a context-dependent sign change in the foreperiod–RT function and orderly accuracy improvements is not straightforward to obtain from a model with fixed inertial parameters alone, although such a model cannot be ruled out. This is more naturally accommodated by interpretations in which passive limb mechanics interact with actively modulated impedance (e.g., co-contraction) to implement context-sensitive triggering strategies. These patterns are also compatible with prior human studies showing context-dependent regulation of limb impedance and co-contraction under uncertainty (Carboni et al., 2021; Börner et al., 2023; Cheng et al., 2025). In this sense, our data provide behavioral signatures that are compatible with MC-inspired accounts while falling short of a mechanistic demonstration of MC.

### 4.4 Limitations and future directions

This proof-of-concept study has several limitations. First, we deliberately adopted a single-participant, high-resolution design. Matching only gross inertial parameters (arm length and mass) across participants would not control the principal sources of between-subject variance because MC depends on the full neuromechanical state, including passive viscoelastic properties and actively modulated impedance (e.g., co-contraction). Premature averaging therefore risks washing out embodiment-specific effects. As a next step, we plan a multi-participant replication to assess generalizability. This design also provided an order-of-magnitude estimate of the effect size; for this participant, the effector-dependent inversion in CV under entropy manipulation fell within a very large range. An *a priori* power analysis using G*Power (effect size = 0.8, α = 0.05, power = 0.95) indicated a required sample size of N = 23. Second, the probabilistic entropy manipulation unequally sampled foreperiod intervals, leaving relatively few trials at longer foreperiods (1.5 s: n = 7; 2.0 s: n = 5 after outlier exclusion). This constraint, inherent to the information-theoretic design, limits power at those intervals; therefore, the apparent deviation at 2.0 s should be interpreted cautiously. Third, we did not strictly control start posture in the reaching movement. This was deliberate: we avoided rigid supports (e.g., levers, elbow rests) and specified only an approximate posture (≈90° elbow flexion) to preserve ecological validity and avoid suppressing morphology-mediated effects. In principle, greater posture variability should inflate trial-to-trial RT variability (i.e., increase CV); yet in our data, CV tended to decrease as prior uncertainty increased, making a simple “posture-noise” account unlikely. While our variability measure targets trial-to-trial initiation variability via CV, slower drifts in timing or strategy may not be fully captured. These warrant future decomposition with richer time-series methods. Finally, our results indicate a phenomenon rather than its mechanism. The observed patterns are compatible with MC, but alternative central-state accounts remain plausible. Attention-based explanations (e.g., reallocating attention across a broader preparatory window under high temporal uncertainty) and strategic adaptations (e.g., within-session learning or predictive planning adjustments) could also yield slower yet less variable responses, reproducing the CV decrease without invoking limb mechanics (Kahneman, 1973; Wolpert and Flanagan, 2001; Howard et al., 2015). To constrain such alternatives, future work should combine concurrent EEG, EMG, and kinematic recordings to track preparation-related activity across the foreperiod and test whether entropy-dependent reductions in CV are mediated by changes in co-contraction or impedance. Even then, EMG must be interpreted cautiously, because co-contraction can decrease with task optimization and learning or increase to improve precision (Thoroughman and Shadmehr, 1999; Gribble et al., 2003). Disentangling these factors will require task designs that control learning across entropy conditions (e.g., counterbalanced block order, within-session entropy randomization), manipulate accuracy demands, and are accompanied by modeling work that incorporates both inertial properties and adaptive impedance control.

## 5 Conclusion

In this proof-of-concept study, we interpreted the results as showing that the mode of embodiment can alter how temporal uncertainty shapes human motor behavior. When the same participant performed low- and high-embodiment movements with foreperiods with manipulated entropy, variability in RT and the foreperiod–RT relationship diverged in a manner consistent with morphology-enabled filtering of uncertainty. These results do not provide a mechanistic demonstration of morphological computation, but they do offer a simple behavioral paradigm for observing and quantifying embodied contributions within the framework of temporal uncertainty. We hope that this basic insight will help to clarify how the brain, body, and environment jointly support robust performance under uncertainty.

## Data Availability

The raw data supporting the conclusions of this article will be made available by the authors, without undue reservation.
