# Supplementary material for: Entropy-dependent human motor modulation consistent with morphological computation in a single subject
Source: Front Robot AI. 2026 Feb 10;13:1734848. doi: 10.3389/frobt.2026.1734848 (PMC12929133; doi:10.3389/frobt.2026.1734848)
Supplement: Supplementary file 1 [file Supplementaryfile1.pdf]

Supplementary Material

Supplementary Table 1. Foreperiod occurrence probabilities by entropy level. Planned probabilities (%) for each foreperiod duration at each entropy level. For example, 0 bit corresponds to a deterministic 1.0 s foreperiod, whereas 2.0 bits corresponds to a uniform distribution; intermediate levels specify graded temporal uncertainty.

**Entropy (bits)**	**Planned probability (%)**			
	0.5 s	1.0 s	1.5 s	2.0 s
0	0	100	0	0
1.0	5	85	5	5
1.5	10	60	20	10
2.0	25	25	25	25

Supplementary Table 2. Experimental sequence, trial counts, and realized entropy by block. For each movement condition (button-pressing, reaching) and planned entropy level (bits), the table shows the execution order of blocks, the number of trials in which each foreperiod (0.5, 1.0, 1.5, 2.0 s) occurred, and the post-calculated entropy for each block. Numbers in parentheses next to the trial counts indicate the number of excluded trials.

**Block sequence**		**Number of trials (excluded)**					**Post-calculated entropy (bits)**
Movement	Entropy (bits)	0.5 s	1.0 s	1.5 s	2.0 s	Total	
Button-pressing	1.5	3 (1)	12	3	2	20 (1)	1.52
Reaching	2.0	7	6 (1)	4	3	20 (1)	1.93
Reaching	0.0	0	20 (1)	0	0	20 (1)	0.00
Button-pressing	2.0	5	5	7 (2)	3	20 (2)	1.97
Reaching	1.5	1	14	2	3 (1)	20 (1)	1.23
Button-pressing	1.0	2	18 (1)	0	0	20 (1)	0.49
Reaching	1.0	1	18 (1)	1	0	20 (1)	0.59
Button-pressing	0.0	0	20 (1)	0	0	20 (1)	0.00
Total		19 (1)	113 (5)	17 (2)	11 (1)	160 (9)	

Supplementary Table 3. Statistical values of regression analysis for the entropy–RT relationship.

Entropy–RT										
**Entropy**	**Movement**	**β0**	**β1**	**n**	**df**	**t**	**p**	**r**	**95% CI (low, high)**	**R²**
Planned	Button-pressing	0.269	0.014	4	2	14.16	0.005	0.995	0.776, 0.999	0.990
	Reaching	0.420	0.024	4	2	10.57	0.009	0.991	0.629, 0.999	0.982
Post-calculated	Button-pressing	0.272	0.013	4	2	5.17	0.035	0.965	0.054, 0.999	0.930
	Reaching	0.425	0.023	4	2	4.25	0.051	0.949	-0.183, 0.999	0.900

Supplementary Table 4. Statistical values of regression analysis for the entropy–CV relationship.

Entropy–CV										
**Entropy**	**Movement**	**β0**	**β1**	**n**	**df**	**t**	**p**	**r**	**95% CI (low, high)**	**R²**
Planned	Button-pressing	8.96	0.19	4	2	0.35	0.760	0.243	-0.937, 0.976	0.059
	Reaching	10.99	-3.34	4	2	-3.41	0.076	-0.924	-0.998, 0.331	0.854
Post-calculated	Button-pressing	9.06	0.11	4	2	0.23	0.839	0.158	-0.947, 0.972	0.025
	Reaching	10.08	-3.05	4	2	-2.02	0.181	-0.820	-0.996, 0.666	0.672

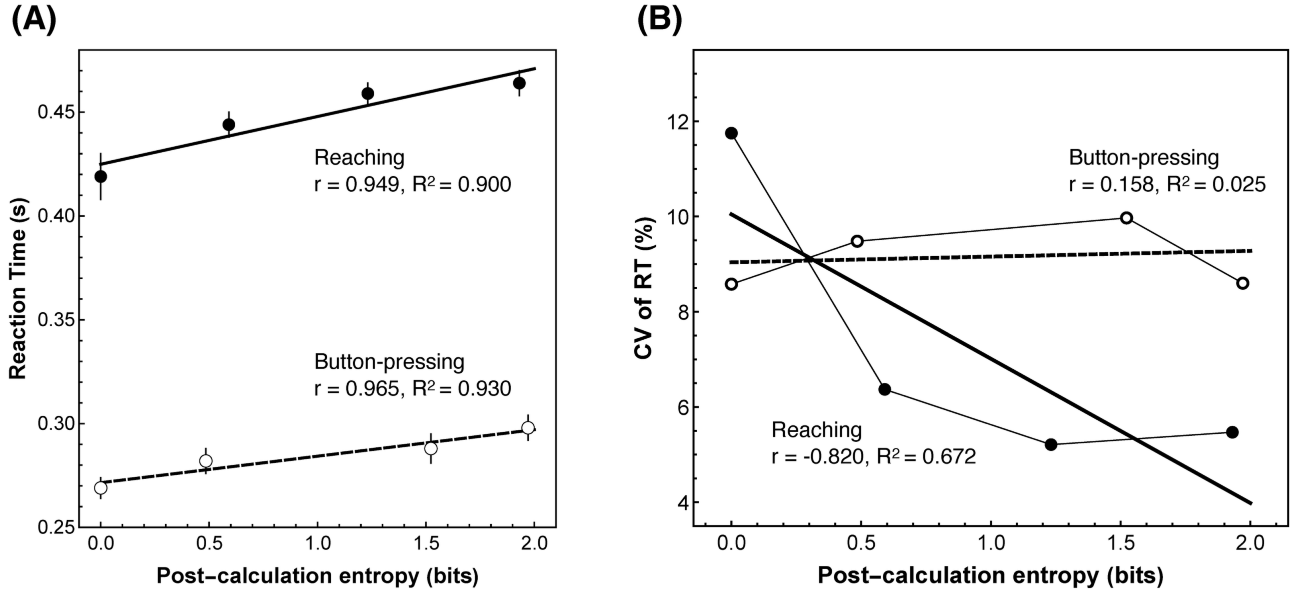

Supplementary Figure 1. Post-calculated entropy analysis of RT and CV. In both figures, black markers indicate reaching condition and white markers indicate button-pressing condition; error bars show standard errors. The solid line is the regression for reaching, and the dashed line is the regression for button-pressing. (A) Correlation between entropy and reaction time (RT). As in the planned-entropy analysis reported in the main text, RT increases with entropy in both movement conditions. (B) Correlation between entropy and the coefficient of variation (CV) of RT. Mirroring

the planned-entropy analysis, button-pressing shows a tendency for CV to increase with entropy, whereas reaching shows a decrease in CV as entropy increases.

Supplementary Table 5. Statistical values of regression analysis for the foreperiod–RT relationship.

Foreperiod–RT

Movement	β0	β1	n	df	t	p	r	95% CI (low, high)	R^2
Button-pressing	0.304	-0.026	4	2	-1.43	0.288	-0.711	-0.993, 0.790	0.505
Reaching	0.393	0.039	4	2	2.11	0.169	0.830	-0.648, 0.996	0.690
